# In vitro study of effects of aging and processing conditions on colour change in maxillofacial silicone elastomers

**DOI:** 10.1186/s12903-019-0798-1

**Published:** 2019-06-19

**Authors:** Ebru Demet Cifter, Meltem Ozdemir-Karatas, Adem Cinarli, Erkan Sancakli, Ali Balik, Gulumser Evlioglu

**Affiliations:** 1 Department of Prosthodontics, Faculty of Dentistry, Istanbul University, 34093 Capa, Istanbul, Turkey; 2 Department of Chemistry, Faculty of Engineering, Istanbul University, 34320 Avcilar, Istanbul, Turkey

**Keywords:** Maxillofacial, Silicone, Elastomer, Colour, Time passage, Dental stone, Dark room

## Abstract

**Background:**

The inherent colour change in maxillofacial silicone elastomers becomes perceptible 6–12 months after fabrication. Determining the factors that accelerate the degradation of the prosthesis can help the clinicians increase its life span. Therefore, the aim of the study was to investigate the effect of time passage, processing temperature, and molding-stone colour on the colour change of maxillofacial silicone elastomers after darkroom storage for 6000 h.

**Methods:**

A total of ten study molds, each incorporating ten specimen gaps were fabricated using five different colors of dental stones. The gaps were filled with coloured Cosmesil M511 maxillofacial silicone elastomer. Five of the study molds, one of each stone color, were processed at room temperature (25 °C) for 24 h while the remainder were vulcanized at 100 °C for 1 h. Two stainless-steel molds were also fabricated to obtain a total of twenty control-group specimens of the same dimensions that were processed under the same conditions as the study molds. Colour measurements of the vulcanized silicone samples were performed using a Konica Minolta spectrophotometer. Initial measurements were obtained after the blocks were removed from the molds and the final measurements were recorded 6000 h after storage in the dark at 25 °C and 40% relative humidity. The CIEDE2000 colour-difference formula was used to measure the changes in the colour. One-way and two-way ANOVA, and an independent-sample t-test were used for statistical assessments.

**Results:**

For every group, the colour change exceeded the perceptible thresholds. Thus, either the vulcanization temperature or the colour of the molding stone has a significant effect on the colour change over time. Those samples vulcanized in green and white molding stones at 100 °C exhibited a significantly higher ∆L*, ∆a*, and ∆b* values relative to the samples vulcanized at room temperature.

**Conclusion:**

The molding-stone colour and vulcanization temperature both affect the degree of colour change after storage in a dark environment. The L*, a*, and b* values for the maxillofacial silicone elastomers are influenced by the direction of the increase or decrease according to the selected colour. This effect varies as the temperature increases.

## Background

Maxillofacial prostheses are used to treat developmental, congenital, or acquired defects in the head and neck region [1]. Unlike dental prostheses, maxillofacial prostheses are preferably fabricated from silicone elastomers which are favoured due to their lifelike appearance and marginal adaptation. However, their long-term colour instability is a problem. Achieving a natural appearance depends on realistic sculpting as well as attaining a good colour match, with the latter being one of the most challenging steps in the manufacture of facial prostheses. Using objective methods in color selection like digital colour selection devices, the researcher can avoid any errors that may result in a subjective evaluation of the observer [2]. However the silicone elastomer is generally coloured by conventional subjective methods as the associated costs are lower. Once the colour is matched, the next challenge is to maintain the colour of the silicone elastomer during the fabrication procedure and throughout the prosthetic’s service life.

The fabrication of a maxillofacial prosthesis consists of a molding phase in which the liquid elastomer becomes an elastic material through a chemical reaction that is known as “vulcanization.” Vulcanization of the maxillofacial prosthesis may be carried out at varying temperatures and for different time periods [3]. Dental stones are commonly used in dentistry are used to fabricate the molds used in this process, as they offer excellent dimensional stability [4].

Minimizing the colour change of a maxillofacial silicone elastomer over time is another challenge, given that the colour change limits the service life of the prosthesis [5]. Although good aesthetic results can be achieved with maxillofacial elastomers, they have to be renewed every 1–2 years because of colour degradation caused by cleansing products, sun exposure, body secretions, and staining of the prosthesis as a result of daily habits [6–12]. The incorporation of nano-oxides, ultraviolet light absorbers, and opacifiers is currently being investigated as a means of enhencing the mechanical properties and colour stability of the silicone elastomers [5, 13–19]. However, colour stability remains a problem for prosthodontists.

Accelerated-aging chambers, outdoor weathering procedures, and darkroom storage methods are all used to estimate the degree of colour change in an elastomer [20]. Lemon et al. have previously evaluated the efficacy of a broad-spectrum ultraviolet light absorber, UV-5411, on the colour stability of a pigmented facial elastomer. The authors concluded that the UV light absorber did not protect the elastomer from colour change but rather accelerated the aging process, causing a greater colour change than outdoor weathering [21]. While the degree of degradation of the mechanical properties of silicone elastomers after darkroom storage remains controversial [22, 23]. Their inherent colour change under these conditions is well-documented [5, 24–29]. The literature lacks comprehensive data on the effect of the vulcanization procedures on the colour change. Determining the factors that accelerate the degradation of the prosthesis can be helpful to the clinicians to increase the life span of the prosthesis and quality of life of the patients who have great aesthetic expectations.

The aim of the present study was to investigate the effects of time passage, processing temperature, and molding-stone colour on the colour change of maxillofacial silicone elastomers after darkroom storage for 6000 h. The null hypothesis of the research was that the molding-stone colour and processing temperature have no effect on the colour of the elastomer over time.

## Methods

**Fig. 1:**
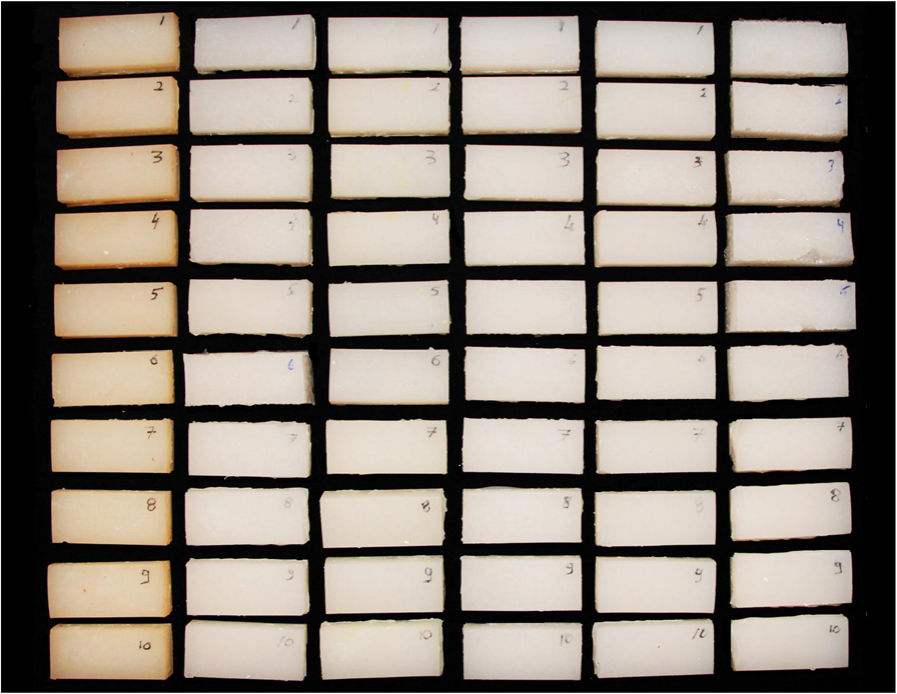
M 511 Maxillofacial silicone elastomer samples

**Fig. 2:**
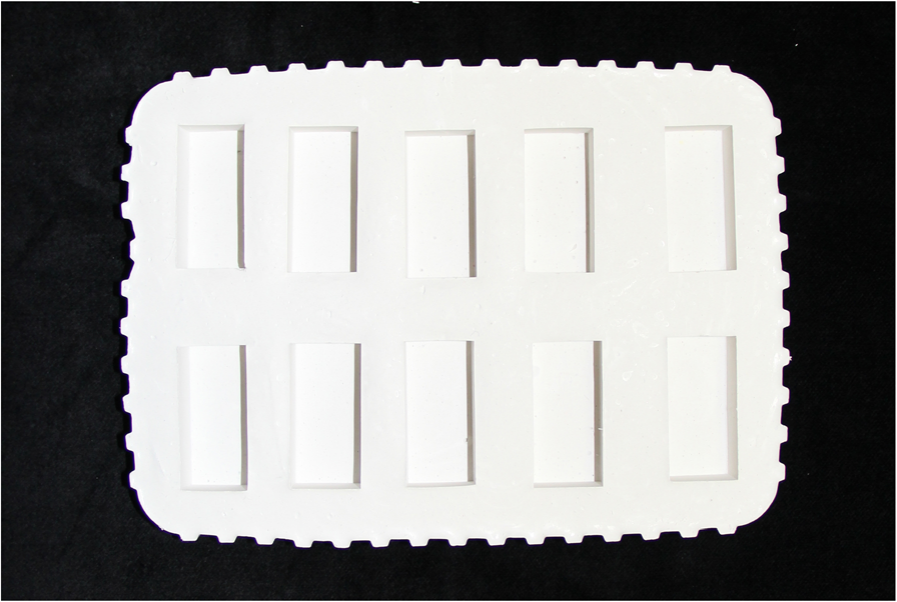
Mold used for vulcanization of white-group samples

**Fig. 3:**
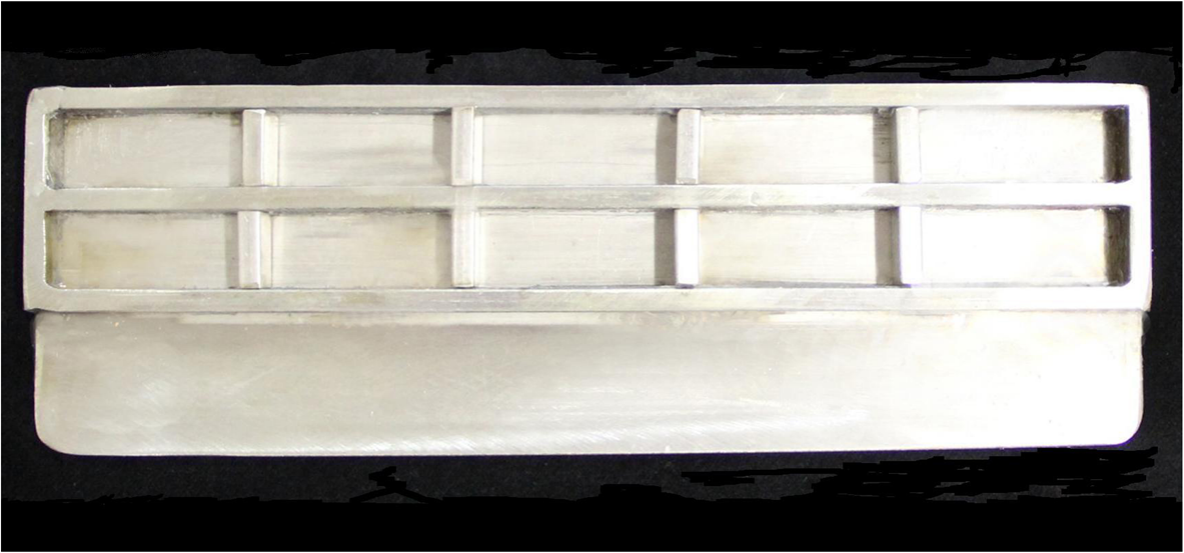
Stainless steel mold fabricated to vulcanize control group samples

**Fig. 4:**
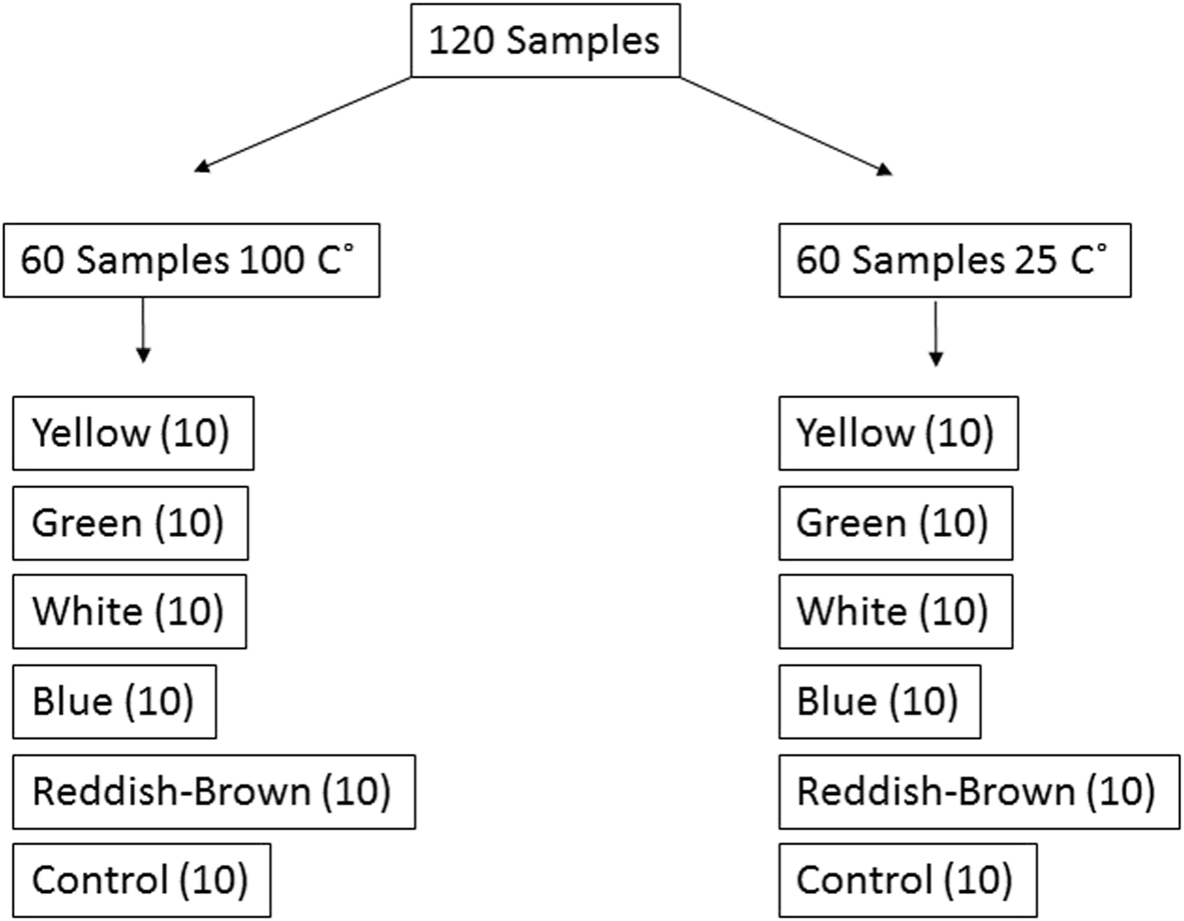
Flow-chart of the study samples

A total of 120 blocks, each measuring 37 × 16 × 10 mm, were fabricated using M511 maxillofacial silicone elastomer (Technovent Ltd., South Wales,UK), at an A:B ratio of 10:1 (Fig. 1). To this, 1.5 g of intrinsic pigments (P105, P108, P112, P410, P413, P414, and P415) were added to attain a fair-skin shade (Fitzpatrick scale III). To prevent any adverse effects on the mechanical and physical properties, the intrinsic pigment weight was kept at less than 0.2% of the elastomer weight, as proposed by Yu et al. [30]. During the fabrication of the elastomer blocks, molding stones of five different colours were used (green: Glastone 3000/Dentsply Inc. York, PA, USA; reddish-brown: Cam-stone N/Ernst-Hinrichs Dental GmbH, Goslar, Germany; white/blue/yellow: Amberok/Anadolu Dental Products, İstanbul, Turkey). The gaps within the molds were formed by embedding 37 × 16 × 10-mm erasers into the molding stone, which was poured into 19 × 13 × 10-mm disposable plastic boxes. Thus, each of the five different-coloured dental stone molds incorporated ten gaps (Fig. 2). Two stainless-steel molds were also fabricated to give a total of 20 control-group specimens of the same dimensions (Fig. 3). A separating medium was applied only to the surface of the molding stone to keep the specimen gaps clear, thus allowing direct contact between the stone and the elastomer. Sixty of the specimens were vulcanized at room temperature (25 °C) for 24 h while the remaining samples were vulcanized at 100 °C for 1 h. The vulcanized silicone samples were washed with an air/water spray for 20 s and then dried for 30 s. The 120 specimens were then separated into twelve groups, namely, room-temperature white, blue, yellow, green, reddish-brown, control/high-temperature white, blue, yellow, green, reddish-brown, and control (Fig. 4).

The initial colour measurements were taken using a spectrophotometer (CM 3600 D, Konica Minolta, Tokyo, Japan) according to the Commission Internationale de l’Eclairage (CIE) colour system. Readings were obtained from three adjacent points on the samples. The instrument was calibrated using a standard white card (L* = 93.3, a* = 0.9, b* = 2.7) before the readings were taken. The same white card was used as the background for the measurements. The L*, a*, and b* values for each specimen were entered into a spreadsheet (Excel 2010, Microsoft Co., Redmond, WA, USA). Here, L* indicates the lightness or darkness, a* is the redness or greenness, and b* is the yellowness or blueness of the specimens. After the initial measurements, all the samples were placed in a chamber and kept in a dark environment at 25 °C and 40% RH for 6000 h. The values of the colour parameters for each specimen after darkroom storage were noted in the same way. The colour change (∆E_00_) was calculated using the following CIEDE2000 equation [31].

$$ {\Delta  \mathrm{E}}_{00}={\left[{\left(\Delta  \mathrm{L}\prime /{\mathrm{k}}_{\mathrm{L}}{\mathrm{S}}_{\mathrm{L}}\right)}^2+{\left(\Delta  \mathrm{C}\prime /{\mathrm{k}}_{\mathrm{C}}{\mathrm{S}}_{\mathrm{C}}\right)}^2+{\left(\Delta  \mathrm{H}\prime /{\mathrm{k}}_{\mathrm{H}}{\mathrm{S}}_{\mathrm{H}}\right)}^2+{\mathrm{R}}_{\mathrm{T}}\ \left(\Delta  \mathrm{C}\prime /{\mathrm{k}}_{\mathrm{C}}{\mathrm{S}}_{\mathrm{C}}\right)\left(\Delta  \mathrm{H}\prime /{\mathrm{k}}_{\mathrm{H}}{\mathrm{S}}_{\mathrm{H}}\right)\right]}^{0.5} $$

The sample size was calculated using the G*Power© v. 3.1.9.2 software (University of Düsseldorf.). A sample size of 10 in each sub-group was needed to achieve a power of 0.80, where α = 0.05, for a hypothetical large effect size (Cohen’s f = 0.40). Statistical analysis were performed using the NCSS 2007 software (NCSS, LLC) to evaluate the effects of time on the colour change and its relationship with the molding-stone colour and vulcanization temperature. Two-way ANOVAs, with Bonferroni corrected post-hoc pairwise comparisons, were applied to test the effects of the vulcanization temperature and molding-stone colour on the degree of colour, L, a and b value changes over time. The statistical significance was set to *p* <  0.05.

## Results

### Effects of colour and temperature on ∆E_00_

A two-way ANOVA was applied to test the effects of vulcanization temperature and molding-stone colour on the degree of colour change that occurs over time. The results were listed in Table 1. These results show that either the vulcanization temperature or the colour of the molding stone had a significant effect on the colour change with time (*p* <  0.001, and *p* = 0.004, respectively). The interaction of these two factors also had a significant effect on the colour change (*p* = 0.001).

**Table 1:** Two-way ANOVA results for colour change (∆E_00_) after 6000 h

Source of variation	Sum of Squares	Df	Mean Square	F	p
Temperature	71.668	1	71.668	20.528	< 0.001*
Colour	66.054	5	13.211	3.784	0.004*
Temperature × colour	75.366	5	15.073	4.317	0.001*
Error	349.127	100	3.491		

*p <  0.05, Df: Degree of freedom, F: Two-way ANOVA

The mean ∆E_00_ scores obtained after 6000 h of dark storage are given in Table 2. The scores for all the samples, including the control group, were in excess of the perceptible thresholds (∆E_00_ = 0.7), as demonstrated by Paravina et al. [32]. The vulcanization temperature was found to have a significant effect on the degree of colour change when yellow and white molding stones were used. Those samples vulcanized in the yellow and white molding stones at 100 °C exhibited a significant colour change after darkroom storage, relative to those samples vulcanized at room temperature (*p* = 0.017, and *p* = 0.007, respectively).

**Table 2:** Mean ∆E_00_ scores and standard deviations representing degree of colour change after 6000 h

Molding Stone Colours	100 °C group	25 °C group	^a^p
	Mean ∆E_00_ ± sd	Mean ∆E_00_ ± sd	
Control	4.11 ± 1.70	2.78 ± 1.87	0.225
Yellow	3.28 ± 1.60	1.71 ± 1.02	0.017*
Green	3.36 ± 1.92	2.39 ± 1.06	0.181
White	6.99 ± 4.52	2.06 ± 1.03	0.007*
Blue	2.84 ± 1.68	1.63 ± 1.03	0.069
Reddish-Brown	3.16 ± 0.61	3.40 ± 1.06	0.542

^a^Independent samples t test, *p <  0.05, ∆E_00:_ Colour change, sd: standart deviation

*P*-values set in boldface indicate statistical significance

### Effects of colour and temperature on ∆L*

The two-way ANOVA analysis was applied to test the effects of vulcanization temperature and molding-stone colour on the ∆L* value over time. The results were listed in Table 3. These results show that either the vulcanization temperature or the colour of the molding stone had a significant effect on the L value change with time (*p* = 0.001, and *p* = 0.006, respectively). The interaction of these two factors also had a significant effect on the L value change (*p* <  0.001).

**Table 3:** Two-way ANOVA results for ∆L*

Source of variation	Sum of Squares	df	Mean Square	F	p
Temperature	121.504	1	121.504	11.219	0.001*
Colour	190.731	5	38.146	3.522	0.006*
Temperature*Colour	397.099	5	79.420	7.333	< 0.001*
Error	1083.035	100	10.830		

*p <  0.05, Df: Degree of freedom, F: Two-way ANOVA

The mean ∆L* scores obtained after 6000 h of dark storage are presented in Table 4. The colour of the molding stone affected the ∆L* value over time when the samples were vulcanized at 100 °C (F: 7.583, *p* <  0.001). The use of green and white molding stones caused the elastomer to whiten over time when vulcanized at 100 °C (*p* = 0.005, *p* = 0.001, respectively). An increase in the vulcanization temperature caused the samples to lighten when they had been formed in yellow, green, or white molding stones (*p* = 0.038, *p* = 0.023, and p <  0.001, respectively). The post-hoc results show that, the samples from the green and white groups lightened significantly relative to the control group (*p* = 0.031,and *p* <  0.001 respectively).

**Table 4:** Comparison of ∆L*values for all study groups

Molding-stone colour	∆L* values	100 °C group	25 °C group	^a^p_diff._
		Mean ∆L* ± sd	Mean ∆L* ± sd	
Control	∆L*	−2.94 ± 4.61	− 0.26 ± 4.01	0.308
	^c^p	0.179	0.879	
Yellow	∆L*	2.10 ± 3.64	−0.92 ± 2.20	0.038*
	^c^p	0.101	0.221	
Green	∆L*	3.37 ± 2.90	0.04 ± 3.10	0.023*
	^c^p	0.005*	0.971	
White	∆L*	7.41 ± 5.16	−1.72 ± 2.21	< 0.001*
	^c^p	0.001*	0.037*	
Blue	∆L*	−0.55 ± 3.65	1.16 ± 1.94	0.209
	^c^p	0.647	0.092	
Reddish-Brown	∆L*	0.73 ± 2.35	−0.90 ± 3.04	0.196
	^c^p	0.349	0.374	
^b^p		F:7.583, p < 0.001*	F: 1.308, p: 0.276	
^‡^Post-hoc		p	p	
Control vs. yellow		0.187	0.999	
Control vs. green		0.031*	0.999	
Control vs. white		< 0.001*	0.999	
Control vs. blue		0.999	0.999	
Control vs. reddish-brown		0.972	0.999	

^a^Independent sample t-test, ^b^One-way ANOVA, ^c^Paired t-test, ^‡^Bonferroni-corrected *p* values,*p <  0.05, sd: standard deviation

*P*-values set in boldface indicate statistical significance

### Effects of colour and temperature on ∆a*

The two-way ANOVA analysis was applied to test the effects of vulcanization temperature and molding-stone colour on the ∆a* value over time. The results were listed in Table 5. These results show that either the vulcanization temperature or the colour of the molding stone had a significant effect on the a* value change with time (*p* = 0.002, and *p* <  0.001, respectively). The interaction of these two factors also had a significant effect on the a* value change (*p* <  0.001).

**Table 5:** Two-way ANOVA results for ∆a*

Source of variation	Sum of Squares	df	Mean Square	F	p
Temperature	0.686	1	0.686	10.036	0.002*
Colour	26.590	5	5.318	77.790	< 0.001*
Temperature × Colour	2.502	5	0.500	7.320	< 0.001*
Error	6.836	100	0.068		

*p <  0.05, Df: Degree of freedom, F: Two-way ANOVA

The mean ∆a* scores recorded after 6000 h of dark storage are presented in Table 6. These results revealed that the colour of the molding stone affects the ∆a* value over time when the samples were vulcanized both at 100 °C and 25 °C (F: 76.177, *p* <  0.001, and F: 46.616, p <  0.001, respectively). The use of green and white stones caused the elastomers to appear redder over time when vulcanized at 100 °C (p <  0.001, and p <  0.001, respectively). The reddening effect was found to be significant for those samples vulcanized in the green and white stones at 100 °C, relative to the 25 °C samples (*p* = 0.018, p <  0.001, respectively). For those samples vulcanized in the reddish-brown molding stones, a significant decrease in the a* values was observed over time, regardless of the temperature (p <  0.001, and p <  0.001respectively).

**Table 6:** Comparison of ∆a*values for all study groups

Molding-stone colour	a*values	100 °C group	25 °C group	^a^p_diff._
		Mean ∆a* ± sd	Mean ∆a* ± sd	
Control	∆a*	− 0.21 ± 0.26	0.03 ± 0.27	0.158
	^c^p	0.110	0.809	
Yellow	∆a*	0.14 ± 0.30	0.05 ± 0.16	0.394
	^c^p	0.166	0.353	
Green	∆a*	0.38 ± 0.19	0.12 ± 0.27	0.018*
	^c^p	< 0.001*	0.204	
White	∆a*	0.72 ± 0.36	0 ± 0.17	< 0.001*
	^c^p	< 0.001*	0.936	
Blue	∆a*	0.05 ± 0.29	0.18 ± 0.19	0.262
	^c^p	0.601	0.016*	
Reddish-Brown	∆a*	− 0.94 ± 0.27	− 1.18 ± 0.32	0.083
	^c^p	< 0.001*	< 0.001*	
^b^p		F: 76.177**,** ***p*** < 0.001*	F: 46.616, p < 0.001*	
^‡^Post-hoc		p	p	
Control vs. yellow		0.313	0.999	
Control vs. green		0.003*	0.999	
Control vs. white		< 0.001*	0.999	
Control vs. blue		0.999	0.999	
Control vs. reddish- Brown		< 0.001*	< 0.001*	

^a^Independent sample t-test, ^b^One-way ANOVA, ^c^Paired t-test, ^‡^Bonferroni-corrected *p* values,*p < 0.05

*P*-values set in boldface indicate statistical significance

Compared to the control group, the increase in the a* value is significant for those samples vulcanized in the green and white molding stones (*p* = 0.003, and p <  0.001 respectively). The decrease in a* over time is significant in the reddish-brown group, relative to the control group, at both temperatures (*p* < 0.001).

### Effects of colour and temperature on ∆b*

The two-way ANOVA analysis was applied to test the effects of vulcanization temperature and molding-stone colour on the ∆b* value over time. The results were listed in Table 7. These results show that the main effect of the colour of the molding stone, and effect of the interaction of the vulcanization temperature and the colour of the molding stone on the “b value change” with time was statistically significant (*p* < 0.001, p < 0.001). The main effect of the vulcanization temperature was not statistically significant (*p* = 0.316).

**Table 7:** Two-way ANOVA results for ∆b*

Source of variation	Sum of Squares	df	Mean Square	F	p
Temperature	0.210	1	0.210	1.016	0.316
Colour	194.455	5	38.891	188.392	< 0.001*
Temperature × Colour	6.879	5	1.376	6.665	< 0.001*
Error	20.644	100	0.206		

*p < 0.05, Df: Degree of freedom, F: Two-way ANOVA

The mean ∆b* scores obtained after 6000 h of dark storage are presented in Table 8. The results reveal that the colour of the molding stone affected the ∆b* value over time when the samples were vulcanized both at 100 °C and 25 °C (F: 109.131, *p* < 0.001*/F: 84.171, p < 0.001*, respectively). The use of the green and white stones caused the elastomers to become yellower over time when vulcanized at 100 °C (*p* = 0.039, and p < 0.001, respectively). For those samples vulcanized in the reddish-brown molding stones, a significant decrease was observed in the b* values over time, regardless of the temperature (p < 0.001, and p < 0.001respectively). Relative to the control group, the increase in the b* value over time for the group vulcanized at 100 °C was significant for those samples vulcanized in the green and white molding stones (*p* = 0.043, and *p* = 0.004, respectively). The decrease in the value of b* over time was significant for the reddish-brown group, relative to the control group, regardless of the vulcanizing temperature (*p* < 0.001). Relative to the control group, the increase in the b* values over time was significant for those samples vulcanized at 25 °C in the yellow molding stones (*p* = 0.044).

**Table 8:** Comparison of ∆b*values for all study groups

Molding-stone colour	b*values	100 °C group	25 °C group	^a^p_diff._
		Mean ∆b* ± sd	Mean ∆b* ± sd	
Control	∆b*	− 0.42 ± 0.69	0.58 ± 0.55	0.020*
	^c^p	0.197	0.051	
Yellow	∆b*	0.10 ± 0.48	− 0.13 ± 0.32	0.226
	^c^p	0.529	0.233	
Green	∆b*	0.35 ± 0.45	0.10 ± 0.27	0.161
	^c^p	0.039*	0.255	
White	∆b*	0.53 ± 0.26	− 0.05 ± 0.25	< 0.001*
	^c^p	< 0.001*	0.531	
Blue	∆b*	0.05 ± 0.40	0.06 ± 0.36	0.937
	^c^p	0.695	0.587	
Reddish-Brown	∆b*	−3.60 ± 0.55	− 3.02 ± 0.73	0.061
	^c^p	< 0.001*	< 0.001*	
^b^p		F:109.131, p < 0.001*	F:84.171, p < 0.001*	
^‡^Post-hoc		p	p	
Control vs. yellow		0.578	0.044*	
Control vs. green		0.043*	0.610	
Control vs. white		0.004*	0.114	
Control vs. blue		0.897	0.417	
Control vs. reddish-brown		< 0.001*	< 0.001*	

^a^Independent sample t-test, ^b^One-way ANOVA, ^c^Paired t-test, ^‡^Bonferroni-corrected *p* values,*p < 0.05

*P*-values set in boldface indicate statistical significance

## Discussion

Changes in the colour and physical properties of maxillofacial prostheses over time require that they be renewed every 6–12 months [22, 24, 25, 33]. The present study evaluated the colour change after 6000 h of darkroom storage, equivalent to 8.5 months of actual service, which approximates to the mean of the time that must elapse before the degradation of the colour of the prosthesis starts. Silicone elastomers are subject to three types of colour change. The first type is the inherent colour degradation that occurs regardless of the colourants, additives, or vulcanization techniques. The second type is the colour change that occurs during the vulcanization stages. The third type is the change that occurs because of external conditions such as the user’s daily habits, cleaning procedures, and the weather [5, 34–36]. Darkroom studies aim to evaluate the first (inherent) type of colour change that occurs as a result of changes to the physical and mechanical properties caused by internal factors within the silicone elastomer chain [3, 37]. An evaluation of the colour change resulting from darkroom storage provides an opportunity to evaluate the colour change due to these internal factors. In the present study, the prolonged effects of the vulcanization procedure were evaluated after darkroom storage.

Hatamleh and Wats have evaluated the effects of the aging conditions on the colour stability of pigmented and non-pigmented elastomers [24]. In their study, a total of 112 samples were divided into five groups that were stored in sebum or acidic perspiration, and then subjected to light aging or outdoor weathering. Control-group samples, including pigmented and non-pigmented samples, were kept in dark storage for six months. The colour change was evaluated by applying the CIELab colour formula. The authors reported that the unpigmented silicone elastomers exhibited inherent colour instability over time, which is in good agreement with the results of the current study. The result of the current study revealed that after 6000 h of dark storage, the mean ∆E_00_ scores for the samples, including the control group, all exceeded the perceptible thresholds (∆E_00_ = 0.7), as demonstrated by Paravina et al. [32]. This indicates that, at both temperatures, the samples vulcanized in the stainless-steel molds underwent an inherent perceptible colour change after 6000 h of darkroom storage.

The colour differences can be measured using the CIELab or CIEDE2000 formulas, as proposed by the Commission Internationale de l’Eclairage. The CIEDE2000 formula used in the present study is a revised version of the CIELab formula. Gómez-Polo et al. have stated that the “CIEDE2000 formula reflected the colour differences perceived by the human eye better than the CIELab formula” [38].

Paravina et al. stated that the perceptibility/acceptability thresholds for fair-skin-coloured maxillofacial silicone elastomers were ∆E_00_ = 0.7/2.1, respectively [32]. In the present study, the colour difference (∆E_00_) scores for all the samples were found to be in excess of the perceptible thresholds. It was concluded that, after 6000 h of dark storage, the colour change becomes perceptible regardless of vulcanization temperature and molding-stone colour. This result provides further evidence for the intrinsic colour degradation of maxillofacial silicone elastomers, which has also been demonstrated by Hatemleh et al., Polyzois et al., Haug et al., and Bankoglu et al. [24, 25, 29, 39].

The intrinsic pigments have also been reported to affect the colour stability over time. Depending on the pigment colour, the ∆E values changed at different levels [13, 14, 28]. Pigments may differ in density, particle size, or morphology, all of which affect their adhesion to the surfaces to which they are applied [40]. Changing the levels of colour transition from molding stone to silicone elastomer have also been reported in the literature [41, 42]. The temperature of the vulcanization process may affect the level of transition by increasing the wettability. When the adhesive forces, caused by intermolecular interactions between a solid surface and a liquid, are higher than the cohesive forces within the liquid, the liquid spreads to the solid surface. The combination of the adhesive forces at the solid–liquid interface and the cohesive forces within the liquid, determines the contact angle. A small contact angle corresponds to a high level of wettability. When the temperature increases, the cohesive forces and the surface tension of the liquid molecules decrease. The adhesive forces cause the solid and liquid molecules to move closer together, thus increasing the wettability [43]. This increase in the contact area may be the reason for the colour transition between the molding stone and the silicone elastomer, as reported by Cifter et al. [41].

The results of the present study reveal that not only the intrinsic factors but also the molding-stone colour and the vulcanization temperature have a significant effect on the colour change after 6000 h. Several studies have reported on the internal colour change of silicone elastomers [24, 25, 29, 30]. However, to the best of our knowledge, the present study is the first to have revealed the long-term effects of the vulcanization temperature and the molding-stone colour on the change in the colour of a silicone elastomer.

In clinical practice, the major and most evident colour change of a maxillofacial prosthesis is the yellowing that occurs over time. The results of the present study indicate that after 6000 h of darkroom storage, this yellowing was much more evident in the control-group samples that were molded in stainless steel and vulcanized at 100 °C (*P* = 0.020). When the green and white molding stones were used together with vulcanization at 100 °C, the yellowing (increase in the b* values) of the elastomer increased significantly after 6000 h (*p* = 0.039, and *p* < 0.001 respectively). It should be noted however that two other studies have claimed that green molding stone is the best option for molding [41, 42]. The first of these studies evaluated the colour degradation immediately after vulcanization, while the second evaluated the colour change of the silicone elastomer molded in stones of three different colours, as well as the effect of three different separating mediums. The ∆b* results of the present study are partially inconsistent with the results of these two studies. Thus avoiding the use of green and white stones for the molding step may extend the service life span of a prosthesis.

This study has several limitations that are important to acknowledge. As the same manufacturer could not supply all colours of dental stones, the study had to be conducted using different brands of dental stones. The pigments that were used to colour the stones during fabrication could have differed from one manufacturer to the other. Furthermore in clinical practice different kinds of separating mediums may be chosen during the molding phase of the maxillofacial prosthesis. In this study design the effect of these mediums was not evaluated in order to monitor the effects of direct contact between the dental stone and the elastomer. Further studies need to be conducted to evaluate the effects of different types of dental stone colorants and separating mediums.

The null hypothesis of the research was: The molding-stone colour and processing temperature have no effect on the colour of the elastomer over time. The null hypothesis was rejected.

## Conclusion

With time there is a perceptible colour change in the maxillofacial silicone elastomers vulcanized in both stainless-steel molds and coloured molding stones. The degree of this colour change is affected by both the vulcanization temperature and the colour of the molding stone.

## Data Availability

The datasets during and/or analyzed during the current study available from the corresponding author on reasonable request.

## Abbreviations

∆E_00_: Colour difference
°C: degrees Celsius
a*: Coordinates of the CIE system indicating redness or greenness
ANOVA: Analysis of variance
b*: Coordinates of the CIE system indicating yellowness or blueness
CIE: Commission Internationale de l’Eclairage
g: Gram
h: Hour
L*: Coordinates of the CIE system indicating lightness or darkness
mm: Millimeter
NCSS: Number Cruncher Statistical System
RH: Relative humidity;
s: Second
SD: Standard deviation

## References

[1] Beumer J, Curtis TA (1996). Maxillofacial rehabilitation: prosthodontics and surgical considerations.

[2] Over LM, Andres CJ, Moore BK, Goodacre CJ, Muñoz CA (1998). Using a colorimeter to develop an intrinsic silicone shade guide for facial prostheses. J Prosthodont.

[3] Ellias HG, Mülhaupt R, Elvers B (2016). Plastics, general survey, 1. Definition, molecular structure and properties. Ulmann’s polymers and plastics: products and process, volume 1.

[4] O’Brien WJ. Dental materials and their selection. 4th ed. Chicago: Quintessence Publishing Company, Inc., pp. 38-62.

[5] Kulkarni RS, Nagda SJ (2014). Colour stability of maxillofacial silicone elastomers: a review of the literature. Eur J Prosthodont Restor Dent.

[6] Craig RG, Koran A, Yu R, Spencer J (1978). Color stability of elastomers for maxillofacial appliances. J Dent Res.

[7] Gary JJ, Huget EF, Powell LD (2001). Accelerated color change in a maxillofacial elastomer with and without pigmentation. J Prosthet Dent.

[8] Polyzois GL (1999). Color stability of facial silicone prosthetic polymers after outdoor weathering. J Prosthet Dent.

[9] Koran A, Powers JM, Lepeak PJ, Craig RG (1979). Stain resistance of maxillofacial materials. J Dent Res.

[10] Yu R, Koran A, Craig RG, Raptis CN (1982). Stain removal from a pigmented silicone maxillofacial elastomer. J Dent Res.

[11] Guiotti AM, Goiato MC, Dos Santos DM, Vechiato-Filho AJ, Cunha BG, Paulini MB (2016). Comparison of conventional and plant-extract disinfectant solutions on the hardness and color stability of a maxillofacial elastomer after artificial aging. J Prosthet Dent.

[12] Yu R, Koran A, Raptis CN, Craig RG (1983). Cigarette staining and cleaning of a maxillofacial silicone. J Dent Res.

[13] Kiat-Amnuay S, Mekayarajjananonth T, Powers JM, Chambers MS, Lemon JC (2006). Interactions of pigments and opacifiers on color stability of MDX4-4210/type a maxillofacial elastomers subjected to artificial aging. J Prosthet Dent.

[14] Kiat-amnuay S, Beerbower M, Powers JM, Paravina RD (2009). Influence of pigments and opacifiers on color stability of silicone maxillofacial elastomer. J Dent.

[15] Akash R. N., Guttal Satyabodh S. (2015). Effect of Incorporation of Nano-Oxides on Color Stability of Maxillofacial Silicone Elastomer Subjected to Outdoor Weathering. Journal of Prosthodontics.

[16] Goiato MC, Haddad MF, Pesqueira AA, Moreno A, Dos Santos DM, Bannwart LC (2011). Effect of chemical disinfection and accelerated aging on color stability of maxillofacial silicone with opacifiers. J Prosthodont.

[17] Han Y, Zhao Y, Xie C, Powers JM, Kiat-amnuay S (2010). Color stability of pigmented maxillofacial silicone elastomer: effects of nano-oxides as opacifiers. J Dent.

[18] Han Y, Powers JM, Kiat-Amnuay S (2013). Effect of opacifiers and UV absorbers on pigmented maxillofacial silicone elastomer, part 1: color stability after artificial aging. J Prosthet Dent.

[19] Tran NH, Scarbecz M, Gary JJ (2004). In vitro evaluation of color change in maxillofacial elastomer through the use of an ultraviolet light absorber and a hindered amine light stabilizer. J Prosthet Dent.

[20] Gary JJ, Smith CT (1998). Pigments and their application in maxillofacial elastomers: a literature review. J Prosthet Dent.

[21] Lemon JC, Chambers MS, Jacobsen ML, Powers JM (1995). Color stability of facial prostheses. J Prosthet Dent.

[22] Hatamleh MM, Polyzois GL, Silikas N, Watts DC (2011). Effect of extraoral aging conditions on mechanical properties of maxillofacial silicone elastomer. J Prosthodont.

[23] Haug SP, Andres CJ, Munoz CA, Okamura M (1992). Effects of environmental factors on maxillofacial elastomers: part III-physical properties. J Prosthet Dent.

[24] Hatemleh MM, Wats DC (2010). Effect of extraoral aging conditions on color stability of maxillofacial silicone elastomer. J Prosthodont.

[25] Polyzois GL, Eleni PN, Krokida MK (2011). Effect of time passage on some physical properties of silicone maxillofacial elastomers. J Craniofac Surg..

[26] Willett ES, Beatty MW (2015). Outdoor weathering of facial prosthetic elastomers differing in durometer hardness. J Prosthet Dent.

[27] Haug SP, Andres CJ, Moore BK (1999). Color stability and colorant effect on maxillofacial elastomers. Part III:weathering effect on color. J Prosthet Dent.

[28] Beatty MW, Mahanna GK, Dick K, Jia W (1995). Color changes in dry-pigmented maxillofacial elastomer resulting from ultraviolet light exposure. J Prosthet Dent.

[29] Haug SP, Andres CJ, Munoz CA, Bernal G (1992). Effect of environmental factors on maxillofacial elastomers: part IV-optical properties. J Prosthet Dent.

[30] Yu R, Koran A, Craig RG (1980). Physical properties of a pigmented silicone maxillofacial material as a function of accelerated aging. J Dent Res.

[31] Sharma G, Wu W, Dalal NE (2005). The CIEDE2000 color-difference formula: implementation notes, supplementary test data, and mathematical observations. Color Res Appl.

[32] Paravina RD, Majkic G, Del Mar Perez M, Kiat-Amnuay S (2009). Color difference thresholds of maxillofacial skin replications. J Prosthodont.

[33] Polzois G, Lyons K (2014). Monitoring shore a hardness of silicone facial elastomers: the effect of natural aging and silicone type after 1 year. J Craniofac Surg.

[34] Griniari P, Polyzois G, Papadopoulos T (2015). Color and structural changes of a maxillofacial elastomer: the effects of accelerated photoaging, disinfection and type of pigments. J Appl Biomater Funct Mater.

[35] Eleni PN, Krokida MK, Polyzois GL, Gettleman L (2013). Effect of different disinfecting procedures on the hardness and color stability of two maxillofacial elastomers over time. J Appl Oral Sci.

[36] Nguyen Caroline Tram, Chambers Mark S., Powers John M., Kiat-amnuay Sudarat (2013). Effect of opacifiers and UV absorbers on pigmented maxillofacial silicone elastomer, part 2: Mechanical properties after artificial aging. The Journal of Prosthetic Dentistry.

[37] Stein RS, Powers J (2006). Topics in polymer physics.

[38] Gómez-Polo C, Portillo Muñoz M, Lorenzo Luengo MC, Vicente P, Galindo P, Martín Casado AM (2016). Comparison of the CIELab and CIEDE2000 color difference formulas. J Prosthet Dent.

[39] Bankoglu M, Oral I, Gul EB, Yilmaz H (2013). Influence of pigments and pigmenting methods on color stability of different silicone maxillofacial elastomers after 1-year dark storage. J Craniofac Surg..

[40] Felton LA, McGinity JW (1999). Influence of pigment concentration and particle size on adhesion of an acrylic resin copolymer to tablet compacts. Drug Dev Ind Pharm.

[41] Cifter ED, Ozdemir –Karatas M, Baca E, Cinarli A, Sancakli E, Gokcen-Rohlig B (2017). Effect of vulcanization temperature and dental stone colour on colour degradation of maxillofacial silicone elastomers. BMC Oral Health.

[42] Sethi T, Kheur M, Coward T, Patel N (2015). Change in color of a maxillofacial prosthetic silicone elastomer, following investment in molds of different materials. J Ind Prosthodont Soc.

[43] Bummer PM (2000). “Interfacial phenomena” Remington's pharmaceutical sciences.

